# The expression of apoptosis-regulating proteins Bcl-2 and Bad in liver cells of C57Bl/6 mice under light-induced functional pinealectomy and after correction with melatonin

**DOI:** 10.18699/VJ21.034

**Published:** 2021-05

**Authors:** S.V. Michurina, I.Yu. Ishchenko, S.A. Arkhipov, A.Yu. Letyagin, M.A. Korolev, E.L. Zavjalov

**Affiliations:** Research Institute of Clinical and Experimental Lymphology – Branch of the Institute of Cytology and Genetics of the Siberian Branch of the Russian Academy of Sciences, Novosibirsk, Russia; Research Institute of Clinical and Experimental Lymphology – Branch of the Institute of Cytology and Genetics of the Siberian Branch of the Russian Academy of Sciences, Novosibirsk, Russia; Research Institute of Clinical and Experimental Lymphology – Branch of the Institute of Cytology and Genetics of the Siberian Branch of the Russian Academy of Sciences, Novosibirsk, Russia; Research Institute of Clinical and Experimental Lymphology – Branch of the Institute of Cytology and Genetics of the Siberian Branch of the Russian Academy of Sciences, Novosibirsk, Russia Institute of Cytology and Genetics of the Siberian Branch of the Russian Academy of Sciences, Novosibirsk, Russia; Research Institute of Clinical and Experimental Lymphology – Branch of the Institute of Cytology and Genetics of the Siberian Branch of the Russian Academy of Sciences, Novosibirsk, Russia; Institute of Cytology and Genetics of the Siberian Branch of the Russian Academy of Sciences, Novosibirsk, Russia

**Keywords:** melatonin, 24-hour lighting, light-induced functional pinealectomy, liver, Bad, Bcl-2., мелатонин, круглосуточное освещение, светоиндуцированная функциональная эпифизэктомия, печень, Bad, Bcl-2

## Abstract

The presence of humans and animals under long-term continuous lighting leads to a suppression of melatonin synthesis, that is, to light-induced functional pinealectomy (LIFP), and the development of desynchronosis. To create LIFP, C57Bl/6 mice were kept under 24-hour lighting (24hL) for 14 days. The animals in the control group were kept under standard lighting conditions. In the next series of experiments, mice with LIFP received daily intragastrically either melatonin (1 mg/kg body weight in 200 μl of distilled water) or 200 μl of water as a placebo. The comparison group consisted of intact animals that received placebo under standard lighting conditions. Immunohistochemical analysis (using an indirect avidin-biotin peroxidase method) revealed the expression of the antiapoptotic protein Bcl-2 and the proapoptotic protein Bad in sinusoid liver cells (a heterogeneous population consisting of the endotheliocytes, Kupffer cells, Ito cells, and Pit cells) and in individual hepatocytes. The Bad expression area in the liver of LIFP mice increased 4 times against a background of the unchanged Bcl-2 expression area. Changes in the brightness (a parameter inversely proportional to the marker concentration) of Bad and Bcl-2 areas did not reach significance. Our results indicate a weakening of the antiapoptotic protection of liver cells of LIFP animals, which creates conditions for activation of the “mitochondrial branch” of apoptosis. Melatonin treatment of LIFP mice resulted in a 3.3-fold increase in Bcl-2 expression area and a 2.7 % decrease in Bcl-2 region brightness compared with the experimental untreated group. Bad protein parameters were unreliable. Thus, melatonin treatment of animals cancels the effect of LIFP, restoring the Bcl-2 expression area and increasing this protein concentration, which indicates an increase in antiapoptotic protection and creates conditions for blocking the development of the “mitochondrial branch” of apoptosis in liver cells.

## Abstract (ru)

Пребывание человека и животных в условиях длительного непрерывного освещения приводит к подавлению синтеза мелатонина, т. е. к светоиндуцированной функциональной эпифизэктомии (СФЭ), и развитию десинхроноза. Для создания СФЭ мыши линии С57Bl/6 содержались в условиях круглосуточного освещения в течение 14 суток. Животные контрольной группы находились при стандартном режиме освещения. В следующей серии экспериментов мыши с СФЭ получали ежедневно внутрижелудочно либо мелатонин (1 мг/кг массы тела в 200 мкл воды), либо в качестве плацебо 200 мкл дистиллированной воды. Группой сравнения служили интактные животные, получавшие плацебо при стандартном режиме освещения. В результате иммуногистохимического анализа (непрямым авидин-биотиновым пероксидазным методом) в синусоидных клетках печени (гетерогенная популяция, состоящая из эндотелиоцитов, клеток Купфера, клеток Ито и pit-клеток) и в отдельных гепатоцитах была выявлена экспрессия антиапоптотического белка Bcl-2 и проапоптотического протеина Bad. В клетках печени мышей с моделью СФЭ обнаружено четырехкратное увеличение площади экспрессии Bad на фоне неизменившейся площади экспрессии Bcl-2. Достоверных изменений яркости (параметр, обратно пропорциональный концентрации маркера) участков, окрашенных на Bcl-2 и Bad, отмечено не было. Полученные данные свидетельствуют об ослаблении антиапоптотической защиты клеток печени животных, содержавшихся при круглосуточном освещении, что создает условия для активации «митохондриальной ветви» апоптоза, наиболее выраженной в синусоидных клетках печени. Введение мелатонина мышам с моделью СФЭ привело к возрастанию в 3.3 раза площади экспрессии Bcl-2 и снижению на 2.7 % яркости (т.е. увеличению концентрации) участков, окрашенных на Bcl-2, по сравнению с опытной группой без лечения. Для Bad изменения исследуемых параметров имели характер тенденций. Таким образом, интрагастральное введение мелатонина животным аннулирует эффект светоиндуцированной функциональной пинеалэктомии, восстанавливая площадь экспрессии и увеличивая концентрацию антиапоптотического белка Bcl-2 в клетках печени, что свидетельствует об усилении антиапоптотической защиты клеток органа и создает условия для блокирования развития «митохондриальной ветви» апоптоза.

## Introduction

At present, human activities are often associated with a change in the natural rhythm of life and with working in artificial lighting conditions, which leads to an increase in the light day period. Lighting at night is considered by scientists as “light pollution”; it is attributed to non-chemical endocrine disruptors affecting both human and animal health, including violations of circadian regulation of melatonin (MT) synthesis, metabolism and other hormone-controlled systems, and the cancer risk (Michurina et al., 2005; Borodin et al., 2012; Russart, Nelson, 2018). By now, scientists have concluded that melatonin is not “a sleep hormone, but a dark hormone” (Reiter et al., 2013; Arendt, 2019). It’s known that light suppresses melatonin production, and darkness weakens this suppression, stimulating the synthesis and release of this hormone into the bloodstream. Of particular importance is the fact that MT suppression in nocturnal rodents is initiated by light. A light pulse lasting only 15 min is sufficient to induce locomotor suppression that endures for more than an hour, and a 1-min light pulse also suppresses MT synthesis for about the same amount of time (Morin, 2013). As a result of long-term stay of humans and animals under 24-hour lighting (24hL) conditions, a decrease/cessation of hormone production leads to the development of light-induced functional pinealectomy (LIFP) (Delibas et al., 2002) and desynchronosis (Reiter et al., 2017; Arendt, 2019). Under these conditions, a significant load falls on the homeostatic systems providing the body resistance (lymphatic, immune and endocrine systems), which are in an integral relationship with the liver, which is the main organ of homeostasis. The study of the structural and functional features of liver cells showed that exactly the cooperative interactions of highly specialized parenchymal liver cells (hepatocytes) and sinusoidal cells (a heterogeneous population of cells consisting of endotheliocytes, Kupffer cells, Ito cells and Pit cells), and their work in a strictly defined rhythm, help the organ to perform numerous functions.

Apoptosis is a fundamental biological mechanism, which causes a clean, non-inflammatory form of cell death and helps the body get rid of unnecessary and defective cells. The ratio of antiapoptotic (Bcl-2, Bcl-XL) and proapoptotic proteins (Bad, Bax, etc.) is considered to be a “molecular switch”, which determines whether tissue growth or atrophy will occur (Willis et al., 2003; Polčic, Mentel, 2020). The features of Bcl-2 family protein expression in liver cells under lightinduced functional pinealectomy remain largely unexplored.

Based on the above, the aim of the study was to evaluate the expression of antiapoptotic Bcl-2 protein and proapoptotic Bad protein in the liver cells of С57Bl/6 mice under light-induced functional pinealectomy and after melatonin treatment.

## Materials and methods

The experiments were carried out in the SPF Vivarium of the Institute of Cytology and Genetics, SB RAS (RFMEFI61914 X0005 and RFMEFI62114X0010). C57Bl/6 mice (male, aged 10–12 weeks) were kept in controlled barrier rooms with free access to water and food (Ssniff, Germany).

Two series of experiments were carried out. In the first event, mice were kept under 24-hour lighting (24hL) for 14 days (light/dark photoperiod 24:0 h) to create light-induced functional pinealectomy (the “24hL” group, n = 6). The comparison group consisted of intact animals (the “Control” group, n = 5) kept under standard lighting conditions (14:10 h). At the same time a smooth increase in illumination to daytime values within 1 hour (dawn) and a smooth decrease in illumination values until complete shutdown within 1 hour (sunset) were assigned to the light phase of the day. In the second series of experiments mice were kept under 24hL for 14 days and received daily intragastrically either melatonin at a dose of 1 mg/kg of body weight in 200 µl of distilled water (the “24hL+MT” group, n = 5) or 200 µl of water (the “24hL+Placebo” group, n = 6). The comparison group consisted of animals (the “Placebo” group, n = 6) kept under standard lighting conditions (14:10 h) and received daily intragastrically 200 µl of distilled water

Animals were removed from the experiment by the craniocervical dislocation method and liver samples were taken for light-optical and immunohistochemical studies. All experiments were performed in accordance with humanity principles and were carried out in compliance with “Rules for working with experimental animals” (The Annex to the Order of the Ministry of Health of the USSR No. 755 of 12.08.1977) and Council Directive 86/609/EEC. Experiments were approved by the local ethical committee (The Protocol No. 128 of 15.03.2017).

Liver samples were fixed in 10 % buffered formalin (BioVitrum, Russia) for 48 hours, dehydrated in a series of alcohols of increasing concentrations and embedded in Histomix (BioVitrum, Russia). Tissue sections with a thickness of 3 μm were prepared on a microtome HM 340Е (Тhermo Fisher Scientific, USA). Immunohistochemical study of the expression of the antiapoptotic Bcl-2 protein and the proapoptotic Bad protein was performed on liver paraffin sections by means of indirect avidin-biotin peroxidase method (ABC-method) using the Vectastain Universal ABC-Peroxidase Kit (Vector Laboratories, Catalog Number PK-7200). At the last stage, immunohistochemical staining was carried out in a chromogenic substrate containing diaminobenzidine (the solution is prepared ex tempore from the components of the set “ImmPACT DAB”; Vector Laboratories, Catalog Number SK-4105).

For quantification of Bcl-2 and Bad expression in the mouse liver, a computer morphometric analysis of digital photographs obtained using a LEICA DM 2500 microscope with a LEICA DFC425C video camera (Germany, Switzerland) at ×400 magnification was performed. The relative area and the brightness of intermediate zones of the hepatic lobules staining for Bcl-2 and Bad were determined in digital images using the program ImageJ. The significance of differences between the compared values was determined using the nonparametric Mann–Whitney test. Differences of compared values were considered statistically significant at p < 0.05.

## Results

**The expression of Bcl-2 and Bad proteins in liver cells of mice under light-induced functional pinealectomy**

**Fig. 1.**
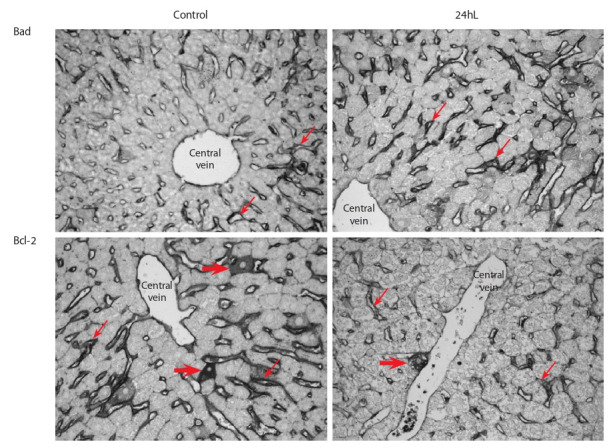
The expression of the proapoptotic Bad protein and the antiapoptotic Bcl-2 protein in mouse liver cells in a light-induced functional pinealectomy model (the “24hL”). Immunohistochemical staining by the indirect ABC method. There is a pronounced Bad coloration and a less pronounced Bcl-2 coloration in sinusoidal cells (thin arrows) of blood sinusoidal capillaries in 24hL mouse liver. Thick arrows indicate separately found stained hepatocytes. Magnification ×400.

A study of Bcl-2 family protein expression in the liver of mice kept under 24-hour lighting (light/dark photoperiod 24:0 h) revealed the pronounced immunohistochemical staining of the proapoptotic Bad protein in sinusoidal cells of blood sinusoid capillaries (Fig. 1). The Bad-positive signal was detected in the endothelium of interlobular veins and in the ductal epithelium of triad bile ducts, and it was also sometimes found in single hepatocytes. At the same time, weak immunohistochemical staining of the antiapoptotic Bcl-2 protein was revealed in sinusoidal liver cells and in single hepatocytes of “24hL” mice liver (see Fig. 1). Staining of Bcl-2 wasn’t determined in the ductal epithelium of triad bile ducts.

**Fig. 2.**
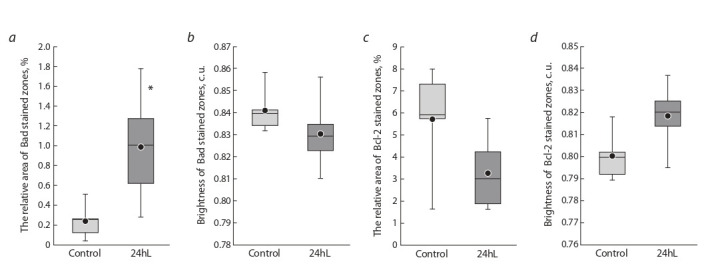
Relative areas of Bad (a) and Bcl-2 (c) protein expressions and the brightnesses of zones stained with these proteins (b – Bad, d – Bcl-2) in the liver of “Control” and “24hL” mice. Notations on the box diagrams: lines – median, boxes – 25–75 %, ● arithmetic mean, * differences are statistically significant between the “Control” and “24hL” groups; the Mann–Whitney U-test (p < 0.05).

Morphometric analysis of liver preparations of the “24hL” animals confirmed the results of the light-optical study. An increase in the Bad expression area was found to be 4.1 times greater than in animals under natural light conditions (Fig. 2, а). At the same time, the brightness (a parameter inverse to the concentration) of the areas stained of that protein did not change significantly (see Fig. 2, b). Changes in the relative area and the brightness of zones stained for the antiapoptotic Bcl-2 protein were in the nature of a trend and reflected a slight decrease in the expression area and concentration of this protein (see Fig. 2, c, d ) in the liver of mice kept under 24-hour lighting.

Thus, it can be concluded that the antiapoptotic protection was weakened and the conditions for apoptosis mitochondrial pathway activation in liver cells of animals with light-induced functional pinealectomy were created.

**Melatonin effect on the expression of Bad and Bcl-2 proteins in mouse liver cells under light-induced functional pinealectomy**

**Fig. 3.**
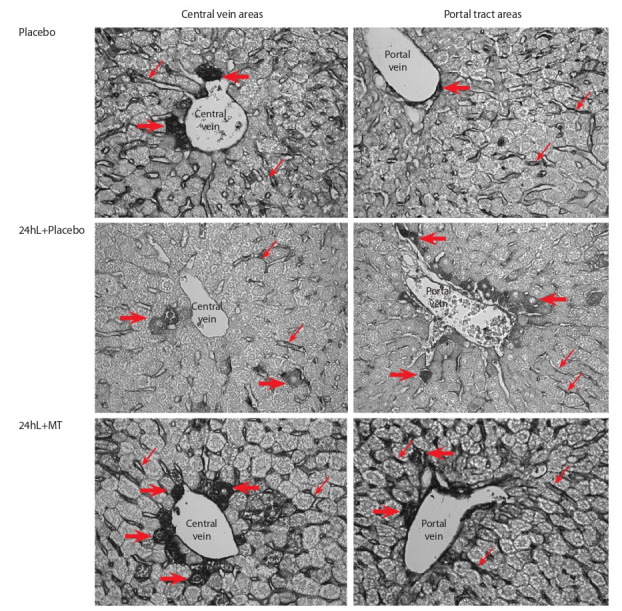
The MT treatment influence on the Bcl-2 expression in liver cells of mice with the LIFP model. Immunohistochemical staining by the indirect ABC method. There is weak Bcl-2-signal in the sinusoidal cells of the liver blood capillaries in “24hL+Placebo” mice compared with “Placebo” animals. MT treatment leads to a pronounced staining of the lining of the blood sinusoidal capillaries, endothelial cells of the portal tract veins, and individual hepatocytes. Thin arrows – the sinusoidal cells, thick arrows – single stained hepatocytes. Magnification ×400.

**Fig. 4.**
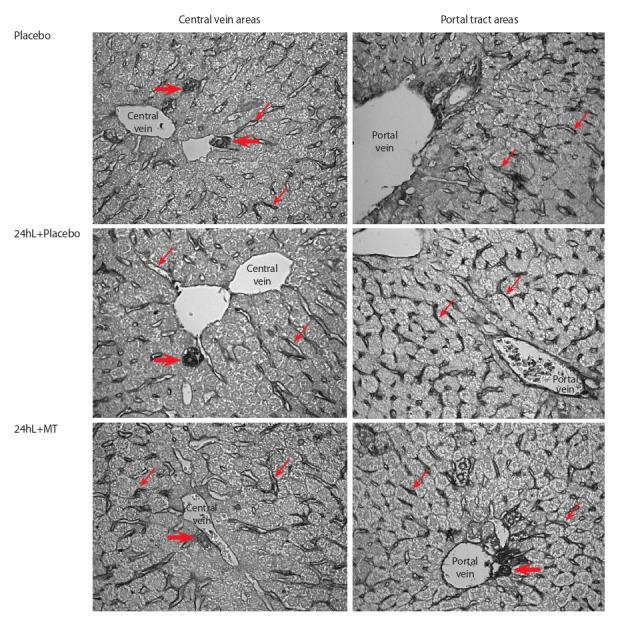
The expression of the proapoptotic Bad protein in liver cells of mice with the LIFP model – in the sinusoidal cells of the liver blood capillaries, the vein endothelium and the ductal epithelium of the bile ducts of the liver portal tracts. Immunohistochemical staining by the indirect ABC method. Bad-staining was more significant in the “24hL+Placebo” group compared to “Placebo”. The Bad expression after MT administration wasn’t as pronounced as Bcl-2 expression (see Fig. 3) in the same animals. Thin arrows – the sinusoidal cells, thick arrows – single stained hepatocytes. Magnification ×400.

MT treatment of the 24hL mice led to the pronounced Bcl-2 protein expression in a heterogeneous population of sinusoidal cells in intra-lobular blood liver capillaries and in single hepatocytes compared to the group without hormone treatment (the “24hL+Placebo” group) (Fig. 3). The immunohistochemical reaction to the Bad protein revealed in all three groups (“Placebo”, “24hL+Placebo”, “24hL+MT”) the staining of sinusoidal capillary lining in intermediate zones and portal tracts, portal vein endothelium and bile duct epithelium in portal tracts (Fig. 4). Bad-staining was more significant in the “24hL+Placebo” group compared to “Placebo”. Bad expression after MT administration wasn’t as pronounced as Bcl-2 expression (see Fig. 3) in the same animals.

**Fig. 5.**
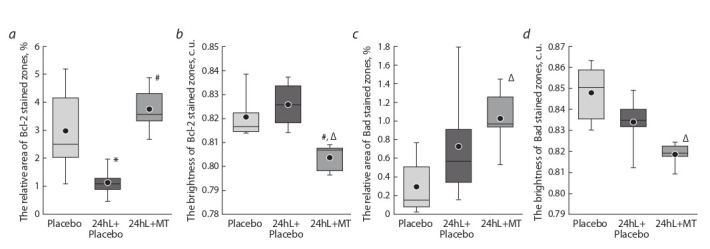
Relative areas of Bcl-2 (a) and Bad (c) protein expressions and the brightnesses of zones stained for these proteins (b – Bcl-2, d – Bad) in the liver of the “Placebo”, “24hL+Placebo” and “24hL+MT” mice. Notations on the box diagrams: lines – median, boxes – 25–75 %, ● arithmetic mean, * differences are statistically significant between the “24hL+Placebo” and “Placebo” groups, # differences are statistically significant between the “24hL+MT” and “24hL+Placebo” groups, ∆ the differences are statistically significant between the “24hL+MT” and “Placebo” groups; the Mann–Whitney U-test (p < 0.05).

Morphometric analysis found a 3.3-fold increase in Bcl-2 expression area in 24hL-animals treated with MT compared with the group without treatment “24hL +Placebo” (Fig. 5, a). At the same time, the studied parameter reached the initial level of the “Placebo” group. The use of MT also led to a significant decrease in brightness (see Fig. 5, b) of stained areas compared with the comparison groups (by 2.7 % – compared with the “24hL+Placebo”, by 2.1 % – compared with the “Placebo”), which reflects an increase in the Bcl-2 concentration in the “24hL+MT” animals. MT intragastric administration contributed to a tendency for an increase in the Bad relative area and a tendency for a decrease in the stained zone brightness compared to animals without hormone treatment. As a result, the use of MT led to a significant increase in the area and concentration of the studied protein compared to the “Placebo” group (see Fig. 5, c, d ).

Thus, MT administration to mice under two-week 24-hour lighting led to a significant increase in the expression area and concentration of the Bcl-2 protein in liver cells against the background of unchanged expression area and concentration of the Bad protein compared to the “24hL+Placebo” group. The obtained results indicate that intragastric administration of MT physiological doses to C57Bl/6 mice cancels the effect of light-induced functional pinealectomy, restoring the expression area of the antiapoptotic Bcl-2 protein and increasing its concentration in liver cells, which indicates increased antiapoptotic protection of organ cells and creates conditions for blocking the apoptosis “mitochondrial branch” development. 

## Discussion

Violation of melatonin production is a starting point, leading at the initial stages to the appearance of desynchronosis followed by the development of organic pathology. Our previous studies showed that 24-hour lighting for two weeks has a modulating effect on all elements of the lymphatic region of the liver. There is a migration of lymphocytes, macrophages into the expanded interstitial non-vascular pathways and lymphatic vessels, and a formation of lymphoid nodules, which are considered temporary accumulations of lymphoid tissue that form in response to injury. The unbalancing of the roots of the lymphatic system leads to the disconnection of contacts between the endothelial cells of the liver sinusoids, as well as to a violation of contacts between the parenchymal cells of the organ. The overflow of Disse spaces with fragments of necrotically altered cells, collagen fibers, lymphoid cells, erythrocytes contributes to the lymph stagnation, and as a result leads to the development of tissue hypoxia, which is an inducer of cell death. This adversely affects the structure and functions of mitochondria, the protein-synthesizing apparatus of cells, causes stress in the endoplasmic reticulum (Ishchenko, Michurina, 2014; Michurina et al., 2018). Under these conditions, a significant burden falls on the intracellular detoxification systems, in particular on the cytochrome P450 system (Woolbright, Jaeschke, 2015). The enzymes of this family can produce reactive oxygen species(ROS), leading to the activation of apoptosis. Excessive and uncontrolled ROS production in mitochondria leads to damage to mitochondrial membranes, proteins, and mitochondrial DNA (mtDNA) and triggers the mitochondrial apoptosis pathway (Li et al., 2020).

In our study, the greatest changes were found in sinusoidal cells of hepatic lobule blood capillaries. This is consistent with the data of Motoyama S. et al. (2000, 2003), who showed the predominant apoptosis development in liver sinusoidal endothelial cells compared to hepatocytes in male SpragueDawley rats with a hypoxia model. Currently, it has been proven that these cells, dynamically regulating the expression of angiopoietin-2, govern their own regeneration, and not only control the proliferation of hepatocytes, but also support the restoration of connective tissue, regulate the maturation and resting state of blood vessels (Hu et al., 2014). Since apoptosis is triggered by the inactivation of Bcl-2 when binding to the Bad protein, the fourfold increase revealed by us in the expression area of the proapoptotic protein Bad against the background of the unchanged expression area of the antiapoptotic protein Bcl-2 in mice with LIFPmodel indicates a decrease in antiapoptotic protection and the apoptosis development along the mitochondrial pathway in liver cells.

It’s found that when melatonin synthesis is disrupted by night lighting, there is a decrease in the activity of its MT1 and MT2 membrane receptors, through which the hormone has its effect on cells (Gupta, Haldar, 2014; Jockers et al., 2016). Due to the non-receptor mechanism using the oligopeptide transporter-1/2 (PEPT-1/2) and organic anion transporter-3 (OAT-3) (Huo et al., 2017) MT penetrates cells and binds free oxygen radicals, protecting macromolecules (proteins, fats, nuclear and mitochondrial DNA) from oxidative damage in all subcellular structures. Currently, numerous data indicate that mitochondria is the main target of MT action: enzymes N-acetyltransferase and hydroxyindole-O-methyltransferase are present in mitochondria and these important subcellular organelles are the place of synthesis of melatonin itself (Hardeland, 2017; Reiter et al., 2018).

There are numerous ways in which MT destroys ROS: starting an antioxidant cascade with the formation of melatonin metabolites detoxifying free radicals; chelating metal ions involved in the Haber–Weiss and Fenton reactions to prevent the formation of a destructive •ОН; stimulating antioxidant and inhibition of pro-oxidant enzymes; increasing the efficiency of electron transfer between mitochondrial respiratory complexes and reducing electron leakage and free radical formation. Studies have shown that MT reduces the rate of apoptosis, prevents the opening of mitochondrial pores and the release of cytochrome c, and preserves mitochondrial functions. In addition, mitochondrial biogenesis and dynamics are also regulated by MT (Hardeland, 2017; Reiter et al., 2018; Jou et al., 2019). The effectiveness of MT as a means of protection against oxidative stress and structural changes in the liver and pancreas tissue was revealed in rats with surgical pinealectomy (Sahna et al., 2004; Col et al., 2010). There is strong evidence that MT has the ability to prevent oxidative damage to liver cell mitochondria in rats with diabetes and obesity (Agil et al., 2015). The question of the effect of this unique hormone on apoptosis is extremely interesting. MT treatment of rats kept under 24-hour lighting during two weeks leads to an increase in the antiapoptotic Bcl-2 protein in the liver (Borodin et al., 2012).

Our use of the melatonin-containing complex in the treatment of animals with a model of obesity and type 2 diabetes mellitus showed its pronounced hepatotropic, lymphotropic action and cytoprotective effect, which consists in stimulating the expression of the antiapoptotic Bcl-2 protein in liver cells against the background of a decrease in the proapoptotic Bad protein activity (Michurina et al., 2017, 2020). In the present study the revealed predominance of the antiapoptotic Bcl-2 protein over the proapoptotic Bad protein, induced by the use of MT, indicates an increase in the antiapoptotic protection of liver cells, which blocks the development of the apoptosis “mitochondrial branch”. This is facilitated by the previously established ability of MT to increase the expression of the lymphatic vascular endothelial LYVE-1 marker in the liver sinusoid endothelial cells of db/db mice, which creates conditions for improving lymph drainage and prevents the development of tissue hypoxia and apoptosis of organ cells (Michurina et al., 2016). The protective properties of MT, largely based on its antioxidant, antiapoptotic, and immunomodulatory activity, place this hormone among the most effective lympho- and angioprotectors (Jing et al., 2017; Chen et al., 2020), which is especially important in the prevention and treatment of new coronavirus infection (Darenskaya et al., 2020; El-Missiry et al., 2020). Thus, melatonin cytoprotective effect revealed by us in the liver cells of C57Bl/6 mice in the model of light-induced functional pinealectomy may be a consequence of reduced damage to mitochondria and other intracellular structures

## Conclusion

Thus our results indicate a weakening of the antiapoptotic protection of liver cells of LIFP animals that creates conditions for activation of the “mitochondrial branch” of apoptosis. Melatonin treatment of animals cancels the effect of LIFP, restoring the Bcl-2 expression area and increasing this protein concentration, which indicates an increase in antiapoptotic protection and creates conditions for blocking the development of the “mitochondrial branch” of apoptosis in liver cells.

## Conflict of interest

The authors declare no conflict of interest.

## References

Agil A., El-Hammadi M., Jiménez-Aranda A., Tassi M., Abdo W.,
Fernández-Vázquez G., Reiter R.J. Melatonin reduces hepatic mitochondrial dysfunction in diabetic obese rats. J. Pineal. Res. 2015;
59(1):70-79. DOI 10.1111/jpi.12241.

Arendt J. Melatonin: countering chaotic time cues. Front. Endocrinol.
(Lausanne). 2019;10:391. DOI 10.3389/fendo.2019.00391.

Borodin Yu.I., Trufakin V.A., Michurina S.V., Shurlygina A.V. Structural and Temporal Organization of the Liver, Lymphatic, Immune,
and Endocrine Systems in Violation of the Light Regime and Melatonin Treatement. Novosibirsk: Manuscript Publ., 2012. (in Russian)
ChenW.R., Yang J.Q., Liu F., Shen X.Q., ZhouY.J. Melatonin attenuates
vascular calcification by activating autophagy via an AMPK/mTOR/
ULK1 signaling pathway. Exp. Cell. Res. 2020;389(1):111883. DOI
10.1016/j.yexcr.2020.111883.

Col C., Dinler K., Hasdemir O., Buyukasik O., Bugdayci G. Oxidative stress and lipid peroxidation products: effect of pinealectomy
or exogenous melatonin injections on biomarkers of tissue damage
during acute pancreatitis. Hepatobiliary Pancreat. Dis. Int. 2010;
9(1):78-82. PMID: 20133234.

Darenskaya M.A., Kolesnikova L.I., Kolesnikov S.I. COVID-19:
oxidative stress and the relevance of antioxidant therapy. Vestnik
Rossijskoj Akademii Meditsynskikh Nauk = Annals of the Russian
Academy of Medical Sciences. 2020;75(4):318-325. DOI 10.15690/
vramn1360. (in Russian)

Delibas N., Tuzmen N., Yonden Z., Altuntas I. Effect of functional pinealectomy on hippocampal lipid peroxidation, antioxidant enzymes
and N-methyl-D-aspartate receptor subunits 2A and 2B in young
and old rats. Neuro Endocrinol. Lett. 2002;23(4):345-350. PMID:
12195239.

El-Missiry M.A., El-Missiry Z.M.A., Othman A.I. Melatonin is a potential adjuvant to improve clinical outcomes in individuals with
obesity and diabetes with coexistence of Covid-19. Eur. J. Pharmacol. 2020;882:173329. DOI 10.1016/j.ejphar.2020.173329.

Gupta S., Haldar C. Nycthemeral variation in melatonin receptor expression in the lymphoid organs of a tropical seasonal breeder Funambulus pennanti. J. Comp. Physiol. A. 2014;200(12):1045-1055.
DOI 10.1007/s00359-014-0959-2.

Hardeland R. Melatonin and the electron transport chain. Cell. Mol.
Life Sci. 2017;74(21):3883-3896. DOI 10.1007/s00018-017-2615-9.

Hu J., Srivastava K., Wieland M., Runge A., Mogler C., Besemfelder E., Terhardt D., Vogel M.J., Cao L., Korn C., Bartels S., Thomas M., Augustin H.G. Endothelial cell-derived angiopoietin-2 controls liver regeneration as a spatiotemporal rheostat. Science. 2014;
343(6169):416-419. DOI 10.1126/science.1244880.

Huo X., Wang C., Yu Z., Peng Y., Wang S., Feng S., Zhang S., Tian X.,
Sun C., Liu K., Deng S., Ma X. Human transporters, PEPT1/2, facilitate melatonin transportation into mitochondria of cancer cells: an implication of the therapeutic potential. J. Pineal Res. 2017;62(4):
e12390. DOI 10.1111/jpi.12390.

Ishchenko I.Y., Michurina S.V. Regional lymph nodes in the liver of
rats in functional pinealectomy. Bull. Exp. Biol. Med. 2014;157(5):
671-676. DOI 10.1007/s10517-014-2636-4.

Jing Y., Bai F., Chen H., Dong H. Melatonin prevents blood vessel
loss and neurological impairment induced by spinal cord injury
in rats. J. Spinal. Cord. Med. 2017;40(2):222-229. DOI 10.1080/
10790268.2016.1227912.

Jockers R., Delagrange P., Dubocovich M.L., Markus R.P., Renault N.,
Tosini G., Cecon E., Zlotos D.P. Update on melatonin receptors:
IUPHAR Review 20. Br. J. Pharmacol. 2016;173(18):2702-2725.
DOI 10.1111/bph.13536.

Jou M.J., Peng T.I., Reiter R.J. Protective stabilization of mitochondrial
permeability transition and mitochondrial oxidation during mitochondrial Ca2+ stress by melatonin’s cascade metabolites C3-OHM
and AFMK in RBA1 astrocytes. J. Pineal Res. 2019;66(1):e12538.
DOI 10.1111/jpi.12538.

Li R., Toan S., Zhou H. Role of mitochondrial quality control in the
pathogenesis of nonalcoholic fatty liver disease. Aging (Albany NY).
2020;12(7):6467-6485. DOI 10.18632/aging.102972.

Michurina S.V., Ishchenko I.Yu., Arkhipov S.A., Cherepanova M.A.,
Vasendin D.V., Zavjalov E.L. Apoptosis in the liver of male db/db
mice during the development of obesity and type 2 diabetes. Vavilovskii Zhurnal Genetiki i Selektsii = Vavilov Journal of Genetics
and Breeding. 2020;24(4):435-440. DOI 10.18699/VJ20.43-o.

Michurina S.V., Ischenko I.Yu., Arkhipov S.A., Klimontov V.V., Cherepanova M.A., Korolev M.A., Rachkovskaya L.N., Zav’yalov E.L.,
Konenkov V.I. Melatonin–aluminum oxide–polymethylsiloxane
complex on apoptosis of liver cells in a model of obesity and type 2
diabetes mellitus. Bull. Exp. Biol. Med. 2017;164(2):165-169. DOI
10.1007/s10517-017-3949-x.

Michurina S.V., Ishchenko I.Yu., Arkhipov S.A., Klimontov V.V.,
Rachkovskaya L.N., Konenkov V.I., Zavyalov E.L. Effects of melatonin, aluminum oxide, and polymethylsiloxane complex on the
expression of LYVE-1 in the liver of mice with obesity and type 2
diabetes mellitus. Bull. Exp. Biol. Med. 2016;162(2):269-272. DOI
10.1007/s10517-016-3592-y.

Michurina S.V., Shurlygina A.V., Belkin A.D., Vakulin G.M., Verbitskaia L.V., Trufakin V.A. Changes in liver and in some organs of immune system of animals exposed to twenty-four-hour illumination.
Morfologiia. 2005;128(4):65-68. PMID: 16400925. (in Russian)

Michurina S.V., Vasendin D.V., Ishchenko I.Yu. Physiological and
biological effects of melatonin: some results and prospects for the
study. Rossiyskiy Fiziologicheskiy Zhurnal im. I.M. Sechenova =
I.M. Sechenov Physiological Journal. 2018;104(3):257-271. (in
Russian)

Morin L.P. Nocturnal light and nocturnal rodents: similar regulation
of disparate functions? J. Biol. Rhythms. 2013;28(2):95-106. DOI
10.1177/0748730413481921.

Motoyama S., Saito S., Alojado M.E., Itoh H., Kitamura M., Suzuki H.,
Saito R., Momiyama H., Nakae H., Ogawa J., Inaba H. Hydrogen
peroxide induces midzonal heat shock protein 72 and apoptosis in sinusoidal endothelial cells of hypoxic rat liver. Crit. Care Med. 2000;
28(5):1509-1514. DOI 10.1097/00003246-200005000-00042.

Motoyama S., Saito S., Saito R., Minamiya Y., Nakamura M., Okuyama M., Imano H., Ogawa J. Hydrogen peroxide-dependent declines
in Bcl-2 induces apoptosis in hypoxic liver. J. Surg. Res. 2003;
110(1):211-216. DOI 10.1016/s0022-4804(03)00006-4.

Polčic P., Mentel M. Reconstituting the mammalian apoptotic switch
in yeast. Genes (Basel ). 2020;11(2):145. DOI 10.3390/genes110
20145.

Reiter R.J., Rosales-Corral S.A., Tan D.X., Alatorre-Jimenez M.,
Lopez C. Circadian dysregulation and melatonin rhythm suppression in the context of aging. In: Jazwinski S., Belancio V., Hill S.
(Eds). Circadian Rhythms and Their Impact on Aging. (Ser. Healthy
Ageing and Longevity. Vol. 7). Springer, Cham, 2017;1-25. DOI
10.1007/978-3-319-64543-8_1.

Reiter R.J., Tan D.X., Rosales-Corral S., Galano A., Jou M.J., AcunaCastroviejo D. Melatonin mitigates mitochondrial meltdown: interactions with SIRT3. Int. J. Mol. Sci. 2018;19(8):2439. DOI 10.3390/
ijms19082439.

Reiter R.J., Tan D.X., Rosales-Corral S., Manchester L.C. The universal nature, unequal distribution and antioxidant functions of melatonin and its derivatives. Mini-Rev. Med. Chem. 2013;13(3):373-384.
DOI 10.2174/1389557511313030006.

Russart K.L.G., Nelson R.J. Light at night as an environmental endocrine disruptor. Physiol. Behav. 2018;190:82-89. DOI 10.1016/
j.physbeh.2017.08.029.

Sahna E., Parlakpinar H., Vardi N., Ciğremis Y., Acet A. Efficacy of
melatonin as protectant against oxidative stress and structural
changes in liver tissue in pinealectomized rats. Acta Histochem.
2004;106(5):331-336. DOI 10.1016/j.acthis.2004.07.006.

Willis S., Day C.L., Hinds M.G., Huang D.C. The Bcl-2-regulated
apoptotic pathway. J. Cell. Sci. 2003;116(Pt.20):4053-4056. DOI
10.1242/jcs.00754.

Woolbright B.L., Jaeschke H. Xenobiotic and endobiotic mediated interactions between the cytochrome P450 system and the inflammatory response in the liver. Adv. Pharmacol. 2015;74:131-161. DOI
10.1016/bs.apha.2015.04.001.

